# Growth Curve, Morphological and Molecular Characterization of Two Strains of *Trypanosoma cruzi* (Kinetoplastida, Trypanosomatidae) isolated from *Triatoma sherlocki* (Hemiptera, Reduviidae, Triatominae)

**DOI:** 10.1590/0037-8682-0521-2021

**Published:** 2022-04-08

**Authors:** Gabriela Kinue Watase Kunii, Rossana Falcone, Leandro da Costa Clementino, João Aristeu da Rosa, Juliana Damieli Nascimento, Tiago Belintani, Jader de Oliveira, Aline Rimoldi Ribeiro

**Affiliations:** 1 Universidade Estadual Paulista “Júlio de Mesquita Filho”, Faculdade de Ciências Farmacêuticas, Araraquara, SP, Brasil.; 2 Universidade Estadual de Campinas, Departamento de Biologia Animal, Campinas, SP, Brasil.; 3 Universidade de São Paulo, Departamento de Epidemiologia, São Paulo, SP, Brasil.; 4 Universidade Estadual de Campinas, Departamento de Genética, Evolução, Microbiologia e Imunologia, Campinas, SP, Brasil.

**Keywords:** Chagas disease, Trypanosoma cruzi, Epidemiology, Vector biology

## Abstract

**Background:**

*Trypanosoma cruzi* presents great variability in morphology, virulence, pathogenicity, avoidance of the host immune system, and antigenic constitution, associated with different clinical manifestations of the disease.

**Methods:**

Two strains of *T. cruzi* were cultivated in liver infusion tryptose to determine growth kinetics, morphometry and molecular characterization using restriction fragment length polymorphism polymerase chain reaction.

**Results:**

The biological parameters showed sharp growth by the 7^th^ day. Morphologically, both strains showed short and thin forms and were classified as Group I.

**Conclusion:**

Group TcI presents cardiac manifestations and *T. sherlocki* is adapting to the home environment, requiring attention to future problems.

Chagas disease is a parasitic infection caused by the flagellate protozoan *Trypanosoma cruzi* (*T. cruzi*) (Chagas 1909)*.* In recent decades, the epidemiological pattern of the disease has changed from rural to urban, affecting people living in metropolitan areas due to population mobility, urbanization, and emigration. Thus, an increasing number of cases have been reported in Canada, the United States of America, Africa, the Eastern Mediterranean, and Western Pacific. Due to the high number of undiagnosed and untreated people, along with areas with active transmission, approximately 75 million people are at risk of infection. During the chronic phase, up to 30% of patients suffer from heart disorders, and up to 10% have digestive, neurological, or mixed disorders. Years later, the infection can lead to sudden death, mainly due to arrhythmia or heart failure caused by the destruction of the heart muscle and its nervous system. Furthermore, patients with Chagas disease are at risk of severe manifestations of coronavirus disease 2019 (COVID-19), Therefore, they should be a priority group to be vaccinated because severe acute respiratory syndrome coronavirus-2 (SARS-CoV-2) can cause myocarditis, and chronic Chagas disease usually leads to a prothrombotic state, cardiac alterations, and secondary thrombotic stroke^1^.

Currently, 18 genera and 157 species of triatomines have been admitted^2^. Among these, 154 species occur in the current era and are potential vectors of *T. cruzi*. However, characteristics such as defecating during or soon after feeding, adaptation to human dwellings, wide geographical distribution, and a high degree of anthropophily^3^ make the genera *Panstrongylus, Rhodnius,* and *Triatoma* the main vectors. *Triatoma sherlocki* (Papa, Jurberg, Carcavallo, Cerqueira & Barata, 2002) is usually associated with rocks and is an endemic specie in Bahia, having medium vector importance. In addition, adults and nymphs infected with *T. cruzi* have been found in the domestic environment of mining communities in the municipality of Gentio do Ouro, indicating that this species is in the process of domiciliation in these areas^4^.

*T. cruzi* presents remarkably high levels of gene diversity and several genetic markers that allow its classification into seven subdivisions. According to the biomarkers large subunit (LSU) 24 Sα-ribosomal deoxyribonucleic acid (rDNA), heat shock protein 60 (*HSP60)*, Histone *H1*, glucose-6-phosphate isomerase (*GPI),* and mini-exon loci using polymerase chain reaction (PCR)^5^, *T. cruzi* strains can be divided into six Discrete Typing Units (DTUs): TcI to TcVI and Tcbat. This molecular characterization is important and contributes to understanding parasite-host interactions and their evolutionary history^5^. Indeed, this molecular complexity reflects on parasite biology, including growth kinetics, antigenic composition, infectivity, and behavior in both vector and mammalian hosts, which emphasizes the importance of characterization of new strains not yet described, thereby contributing to increasing the existing knowledge about *T. cruzi* and its genetic variability.

In this study, the Tsh 4 and 18 *T. cruzi* strains were isolated from nymphs and adult feces of *Triatoma sherlocki* captured in Santo Inácio, the municipality of Gentio do Ouro, Bahia. Growth kinetics studies were performed in triplicate by cultivating 1.5 × 10^6^ parasites/mL in liver infusion tryptose (LIT) to evaluate the growth rate of the parasite in the first days of culture^6^ ^,^ ^7^. In addition, epimastigote forms were counted in the four outer quadrants of the Neubauer chamber under an optical microscope for 10 consecutive days.

According to the manufacturer’s instructions, for molecular characterization, *T. cruzi* genomic deoxyribonucleic acid (DNA) was extracted using the PureLink™ Genomic DNA Mini Kit (Invitrogen™). The Tsh 4 and 18 *T. cruzi* strains were characterized using PCR amplification of the D7 divergent domain of the 24 Sα ribosomal ribonucleic acid (rRNA) gene (LSU rDNA), according to Souto and colleagues (1996)^8^, and the products were separated by electrophoresis in 3% agarose gel stained with ethidium bromide. The amplification of the genes *HSP60* and *GPI* was performed using restriction fragment length polymorphism polymerase chain reaction (RFLP-PCR) with the following target/restriction enzyme combinations: *HSP60*/*EcoRV* and GPI/HhaI and incubated for 4 h at 37 ºC. The length and restriction profiles of the amplified *HSP60* and *GPI* genes were analyzed by electrophoresis in 1-3% agarose gels stained with ethidium bromide^5^.

Morphological characterization was performed using images analyzed under a light microscope (Olympus, BX-51) at x 1,000 using 30 epimastigotes from LIT and 30 trypomastigotes from artificial urine triatomine (TAU) of both strains. The morphological parameters used were total parasite length, body length, free flagellum, width, kinetoplast area, nucleus area, distance from anterior extremity to the nucleus, distance from posterior extremity to the nucleus, and nuclear index (IN=PN/NA) for trypomastigote forms, and width, total length, nucleus area, and kinetoplast area for epimastigote forms^8^.

The growth of *T. cruzi* strains was verified during epimastigote multiplication in LIT medium, according to the results presented in Figure 1. 

**FIGURE 1:**
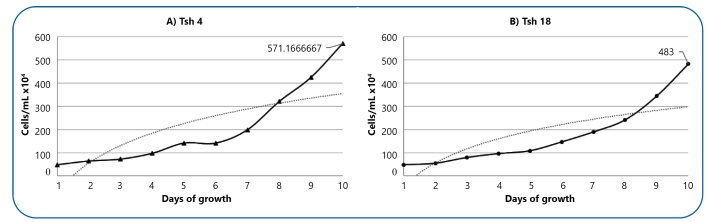
Growth kinetics of the Tsh 4 and 18 *T. cruzi* epimastigote strains in LIT. Black line represents linear scale. Gray line represents logarithmic scale.

The product of the amplified *HSP60* gene presented a 290 bp band, the *GPI* gene presented the formation of two bands (400 bp and 240 bp), and the LSU rDNA gene presented a 110 bp band gel for both strains (Figure 2). These results are in accordance with those reported in the literature and suggest the classification of *T. cruzi* into the Tsh 4 and 18 strains belonging to the TcI group. In addition, the peak growth of the parasite on the 10th day of infection was also characteristic of the TcI strains.

**FIGURE 2:**
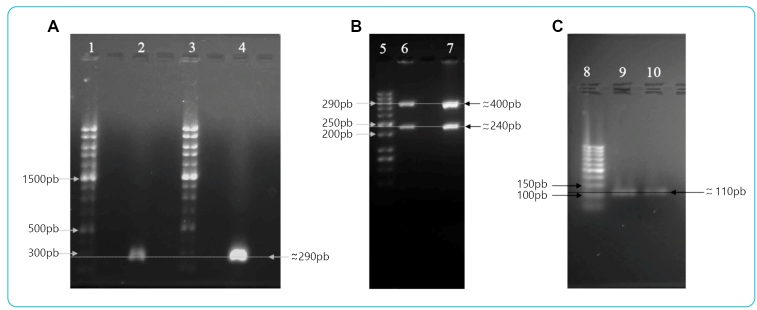
Genotyping of *Trypanosoma cruzi* isolates using PCR assays based on LSU rDNA; *HSP60* and *GPI*. **(A)** PCR-RFLP genotyping profiles. *HSP60* gene digested. Lanes: *1* and *3,* molecular weight 1Kb Plus (Invitrogen); *2,* Tsh 4 strain; *4,* Tsh 18 strain. **(B)** PCR-RFLP genotyping profiles. *GPI* gene digested products. Lanes: *5,* molecular weight 50 pb DNA Ladder (Cellco); *6,* Tsh 4 strain; *7,* Tsh 18 strain. **(C)** LSU rDNA PCR product size polymorphism genotyping assay profiles. Lanes: *8,* molecular weight 50 pb DNA Ladder (Cellco); *9,* Tsh 4 strain; *10,* Tsh 18 strain.

The results of the morphometric evaluation of 30 epimastigote and 30 trypomastigote forms of the Tsh 4 and 18 *T. cruzi* strains are presented in Table 1.

**TABLE 1:** Results of morphometric evaluation of 30 epimastigote and 30 trypomastigote forms of the Tsh 4 and 18 *Trypanosoma cruzi* strains. (x¯ ± SD) = average ± standard deviation.

Morphometric Parameters	Tsh 4						Tsh 18					
	Epimastigote (µm)			Trypomastigote (µm)			Epimastigote (µm)			Trypomastigote (µm)		
	Min.	Max.	**x¯ ± SD**	Min.	Max.	**x¯ ± SD**	Min.	Max.	**x¯ ± SD**	Min.	Max.	± SD
Width	1.32	2.42	1.81 ± 0.25	0.57	1.94	1.29 ± 0.32	1.06	2.16	1.67 ± 0.24	1.07	1.95	1.38 ± 0.23
Total length	14.84	27.46	21.41 ± 3.52	13.00	26.75	19.40 ± 3.21	14.77	28.21	20.39 ± 3.60	12.81	22.89	18.31 ± 2.53
Core area	0.92	2.80	1.87 ± 0.53	1.61	3.93	2.27 ± 0.52	1.08	2.58	1.54 ± 0.37	1.19	3.14	2.52 ± 0.39
Kinetoplast area	0.59	1.55	0.97 ± 0.25	0.56	1.99	1.06 ± 0.30	0.40	1.60	0.80 ± 0.23	0.63	1.46	1.05 ± 0.18
Free length of the flagellum	-	-	-	3.60	9.57	6.02 ± 1.50	-	-	-	3.11	9.49	6.57 ± 1.73
Body length	-	-	-	7.88	17.78	13.39 ± 2.80	-	-	-	7.21	16.15	11.73 ± 1.99
Distance from pre-core end (NA)	-	-	-	7.42	19.97	12.55 ± 3.11	-	-	-	9.11	15.83	12.72 ± 2.12
Distance from posterior end to core (PN)	-	-	-	3.43	10.88	6.79 ± 1.69	-	-	-	3.56	9.46	5.57 ± 1.28
Nuclear content (IN=PN/NA)	-	-	-	0.27	1.06	0.58 ± 0.24	-	-	-	0.30	0.97	0.45 ± 0.14

DNA analyses of Tsh 4 and 18 strains fit as Group I of *T. cruzi,* since they presented a 110 bp band as a product of amplification of the LSU rDNA gene, one band for the HSP60 gene, and two bands of the GPI gene^5^. The TcI group is the most prevalent DTU group, from which four isolated genotypes have been identified (TcIa-TcId). The Id group is present in South America and is associated with the wild cycle of Chagas disease, characterized by a deletion of nine nucleotides at positions 15-23 of the microsatellite region of the mini exon gene^9^. 

To contribute to the morphological characterization of the Tsh 4 and 18 *T. cruzi* strains*,* they were measured and classified according to Rimoldi et al. (2012)^6^, where the epimastigotes were mostly thin (56% Tsh 4 and 70% Tsh 18), short (66% Tsh 4 and 76% Tsh 18), with an intermediate core area (63%) for the Tsh 4 strain and small (70%) for the Tsh 18 strain, as well as an intermediate kinetoplast area (50% Tsh 4 and 80% Tsh 18). In contrast, the trypomastigotes were mostly short (53% Tsh 4 and 73% Tsh 18), with short flagella (80% Tsh 4 and 66% Tsh 18), thin (66% Tsh 4 and 50% Tsh 18), area of the kinetoplast mostly intermediate (63% Tsh 4 and 76% Tsh 18), small core area (53%) for the Tsh 4 strain, intermediate (76%) for the Tsh 18 strain, and 100% of the low nuclear index.

As demonstrated by Silva (1959)^10^ and Rimoldi et al. (2012)^6^, although dimorphism is present to a greater or lesser extent in some strains, one form is predominant. However, the incidence of this variability still lacks studies, and it is unknown whether it reflects a difference in biological behavior between strains.

The *in vitro* behavior of strains obtained from human cases and *Triatoma infestans* in the LIT medium differed by Brener and Chiari (1965)^7^. When researching the behavior of strain Y in the LIT medium, Chiari (1974) discovered a 4-day development phase. The number of epimastigotes grew slowly in the late exponential phase before reaching a plateau. The Tsh 4 (5.71 × 10^6^ parasites/mL) and 18 (4.83 × 10^6^ parasites/mL) *T. cruzi* strains displayed a growth phase on the 7^th^ day, related to the parasite group TcI. A typical growth curve was observed with two distinct phases: log or exponential, and early stationary. From days 1 to 5, the log phase is defined by an exponential growth rate, whereas the early stationary phase is characterized by a significant decrease in growth rate (6-10 days)^11^. These results are expected, and we consider it useful to compare them with other *T. cruzi* strains and help to improve the existing data.

In combination, these results suggest that the maintenance of *T. cruzi* I populations may be related to intrinsic characteristics of the parasite, such as kinetics of growth, and reinforce the importance of studying *T. cruzi* isolated from natural hosts.

According to the Epidemiological Bulletin of Chagas Disease in the State of Bahia (2021)^12^, Chagas disease is highly expressed, with an annual average of 621 deaths from 2010 to 2019. The mortality rate makes Bahia the fourth highest among federated units. The counties with the highest rates per 100,000 inhabitants are Contendas do Sincorá (49.2), Dom Macedo Costa (49.3), Mulungu do Morro (55.1), and Angical (71.5). In addition, the municipality of Gentio do Ouro, where specimens of triatomine infected with Tsh 4 and 18 strains were collected, belonged to the regional health Irecê, which is included in the municipalities with higher risk rates starting at 20.0/100,000.

To date, the presence of *T. sherlocki* has only been described in the district of Santo Inácio, Bahia. Although the species is considered wild, there are reports of its presence in homes, which may indicate that adaptation to the home environment is taking place^13^ ^,^ ^14^. This situation becomes worrisome because of the capture of specimens infected with *T. cruzi,* which could result in vector transmission to the residents, a possibility that requires constant vigilance since infections by the TcI group present cardiac manifestations. In addition, the specimens used in this study came from the same area where *T. sherlocki* was collected and described, and both were found to be infected with *T. cruzi,* which proves that the epidemiological cycle of the disease persists throughout the region^15^. 
